# An interpretable machine learning model for diagnosis of Alzheimer's disease

**DOI:** 10.7717/peerj.6543

**Published:** 2019-03-01

**Authors:** Diptesh Das, Junichi Ito, Tadashi Kadowaki, Koji Tsuda

**Affiliations:** 1 Department of Computational Biology and Medical Sciences, Graduate School of Frontier Sciences, The University of Tokyo, Chiba, Japan; 2 Data Science Laboratory, hhc Data Creation Center, Eisai Co. Ltd., Tsukuba, Japan

**Keywords:** Dementia, Interpretable model, Sparse high-order interaction, Alzheimer’s disease (AD), Computer-aided diagnosis (CAD) model, SHIMR, ADNI, Cost-effective framework, Machine learning model, Classification with rejection option

## Abstract

We present an interpretable machine learning model for medical diagnosis called sparse high-order interaction model with rejection option (SHIMR). A decision tree explains to a patient the diagnosis with a long rule (i.e., conjunction of many intervals), while SHIMR employs a weighted sum of short rules. Using proteomics data of 151 subjects in the Alzheimer’s Disease Neuroimaging Initiative (ADNI) dataset, SHIMR is shown to be as accurate as other non-interpretable methods (Sensitivity, SN = 0.84 ± 0.1, Specificity, SP = 0.69 ± 0.15 and Area Under the Curve, AUC = 0.86 ± 0.09). For clinical usage, SHIMR has a function to abstain from making any diagnosis when it is not confident enough, so that a medical doctor can choose more accurate but invasive and/or more costly pathologies. The incorporation of a rejection option complements SHIMR in designing a multistage cost-effective diagnosis framework. Using a baseline concentration of cerebrospinal fluid (CSF) and plasma proteins from a common cohort of 141 subjects, SHIMR is shown to be effective in designing a patient-specific cost-effective Alzheimer’s disease (AD) pathology. Thus, interpretability, reliability and having the potential to design a patient-specific multistage cost-effective diagnosis framework can make SHIMR serve as an indispensable tool in the era of precision medicine that can cater to the demand of both doctors and patients, and reduce the overwhelming financial burden of medical diagnosis.

## Introduction

Alzheimer’s disease (AD) is a progressive disease affecting memory and other mental functionalities with deteriorating symptoms over time. With increased human life expectancy, a large number (11–16 million) of elderly people are likely to suffer from AD by 2050 (Alzheimer’s Association, 2015). Treatment of AD is often hampered due to the lack of easily accessible and cost-effective biomarkers with reliable diagnostic accuracy. To counter this problem, the Alzheimer’s Disease Neuroimaging Initiative (ADNI) study began in 2004 with the intention of collecting and storing a multitude of data spanning across clinical data, imaging data, omics, gene expression data, etc. Fluid based biomarkers, for example, cerebrospinal fluid (CSF) and neuroimaging such as magnetic resonance imaging (MRI) or positron emission tomography (PET) are highly accurate, but often not feasible for clinical implementation due to either their high cost, invasive nature, or lack of specialized clinics offering such services. Consequently, effective treatments are only available to limited patients. These limitations have significant impact on both the patients lacking effective treatment for AD as well as the health care system trying to cope with such a substantial financial burden (Henriksen et al., 2014). Therefore, the goal of ADNI core research (Henriksen et al., 2014) is to find a cost-effective way (e.g., blood based biomarkers or cognitive assessment) that can serve as the first step in a multistage diagnostic or prognostic process followed by most advanced and expensive pathologies such as CSF or MRI screening. Another important aspect to this issue is that it is not feasible for a medical practitioner, even as an expert in this domain, to exploit such a vast and diverse datasets manually. Hence, there exists an urgent need of advanced computer-aided diagnosis (CAD) framework that can serve as a helping hand to medical practitioners to better understand the disease and design a patient-specific medical regime. A line of research has been conducted to devise such CAD methods. Deep learning based automated diagnosis framework (Fisher, Smith & Walsh, 2018; Lu et al., 2018; Charan, Khan & Khurshid, 2018) and hyper spectral imaging based methods (Khan et al., 2018) are examples of state-of-the-art CAD methods. However, it is often the case that a medical practitioner cannot rely on state-of-the-art CAD methods despite its high accuracy. Due to the fact that most of these methods are opaque and cannot answer these basic question: why/how has it reached such a decision and why/how is it biologically relevant? (Freitas, Wieser & Apweiler, 2010; Freitas, 2006; Burrell, 2016; Ribeiro, Singh & Guestrin, 2016b). Recently, the European Union has issued a “general data protection regulation (GDPR)” on algorithmic decision-making and a “right to explanation” (Goodman & Flaxman, 2016) which mandates that the data subject has the right to “meaningful information about the logic involved in the decision making.” In other words, the GDPR requires that communication with data subjects has to be made in a “concise, intelligible, and easily accessible form.” Therefore, to cater to the demand of both, the medical practitioner (doctor) and the subject (patient), the most effective approach would be to design a cost-effective multistage CAD framework where the trained model can be articulated and easily understood by a human. In a sense the model should provide enough information about how input features relate to predictions and allow one to answer questions such as: Is the prediction biologically relevant? Which features play the most important role in prediction? Why am I diagnosed as diseased/normal? Why the prescribed treatment is the optimum one given my current medical condition? For these reasons, existing CAD methods involving rule induction algorithms such as propositional rules, decision tables, decision trees (DT) etc. (Huysmans et al., 2011; Kim, Rudin & Shah, 2014; Wang & Rudin, 2015) are often preferred with the aim of generating an interpretable set of “if_then” rules. A line of research (Ribeiro, Singh & Guestrin, 2016c, 2016a; Zhou, Jiang & Chen, 2003) has also been conducted to extract set of human understandable rules from black box models (neural networks, support vector machines (SVM) etc.). However, previous research (Huysmans, Baesens & Vanthienen, 2006; Johansson, Konig & Niklasson, 2005) on rule induction algorithms and post hoc rule extraction methods either suffer from very complex rule generation or result in a suboptimal set of decision rules owing to the nature of optimization problems formulated in their computational model. Another longstanding concern of a domain expert wanting to embrace a rule based computation model is its poor representation, as highlighted by Pazzani (2000) that has had very little effort invested to empirically assess interpretabilty beyond simply reporting the size of the resulting representations. Pazzani (2000) also highlighted that there have been no attempt of visual representation, whereas users prefer certain visualization over mere textual or graphical description. To address the above issues, we present a *“sparse high-order interaction model with rejection option”* (SHIMR).

**Figure 1:**
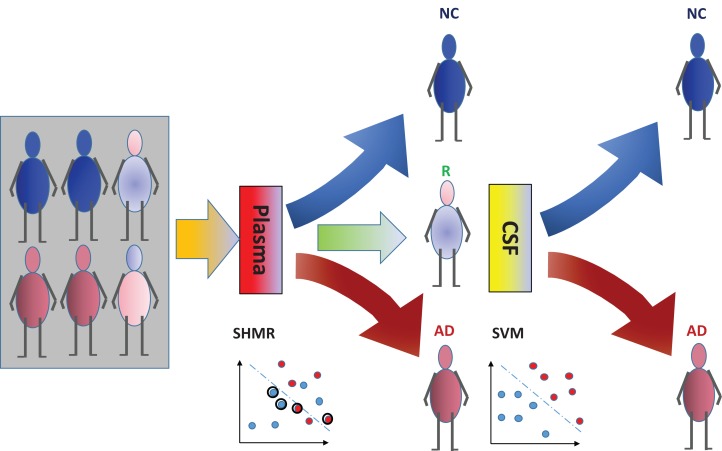
Cost-effective multistage framework for the diagnosis of Alzheimer’s disease (AD) patients from normal control (NC). In a clinical setting, all registered patients can undergo an initial screening using inexpensive and easily accessible biomarkers (e.g., Plasma). Only those patients difficult to diagnosis (hence “Rejected: R” by SHIMR) are recommended for invasive and/or more expensive screening (e.g., CSF). Abbreviation: CSF, cerebrospinal fluid; SVM, support vector machines.

**Figure 2:**
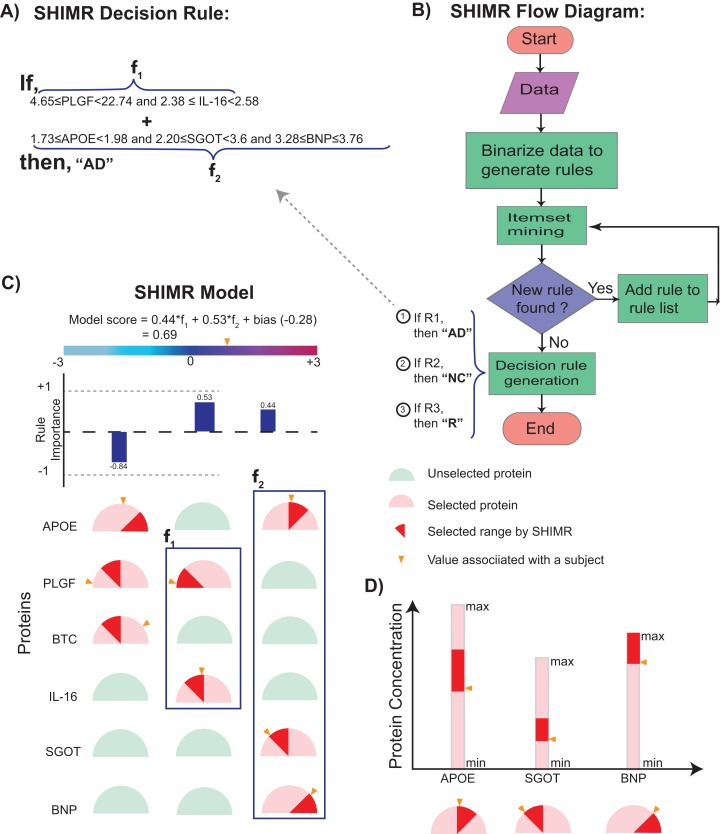
Visualization of SHIMR. The selected rules (A), which are generated by SHIMR (B), are described as an intersection matrix (C). Each row in the intersection matrix (C) represents individual protein and each column represents interaction among proteins constituting a rule. Proteins selected by a rule are represented by “light red gauge” (semicircles), whereas the unselected ones by “light green gauge.” The exact selected range of a particular protein is highlighted by “dark red wedge.” The blue rectangular box surrounding a set of proteins highlights the selected protein combination (or feature) for a subject. Blue colored bars above each column show the importance of each rule contributing to the overall “model score.” The generated model score for a subject (patient) is also highlighted by an “orange pointer” over a color bar at the top of each plot. This color bar describes the overall range of model scores. Construction of the intersection matrix from different concentrations of individual proteins is also shown (D). Each gauge represents the concentration range of a particular protein where the left end represents the minimum and the right end represents the maximum. The “orange pointer” over each protein gauge describes the exact value of protein concentration corresponding to a particular subject. Abbreviations: NC, normal control; AD, Alzheimer’s disease; R, rejected; R1, rule 1; R2, rule 2; R3, rule 3; f1, feature 1 of rule 1; f2, feature 2 of rule 1.

The incorporation of rejection option complements SHIMR to design a multistage cost-effective framework (Fig. 1) with the notion of refraining from making any decision (Reject them: R) for those data patients which are hard to classify (patients who are close to the decision boundary of a CAD model) and make prediction for only those patients for which the model is confident enough (patients which are far apart from the decision boundary of a CAD model). Patients rejected due to the low confidence of a first stage model, trained on inexpensive and easily accessible biomarkers (e.g., plasma) can further be recommended for second stage of evaluation, involving advanced and complex screening (e.g., CSF). However, medical practitioners must be able to interpret the model and identify the variables separately for rejected and classified samples and decide the subsequent course of treatment accordingly. To address that, SHIMR employs interval conjunction rules that are highly interpretable as well as accurate decision sets. Decision sets which consist of sets of “if-then” rules are in general simple, concise and highly interpretable (Lakkaraju, Bach & Leskovec, 2016) as shown in Fig. 2, which was generated by our visualization module. Another potential advantage of using interval conjunction rules is that it can capture the combinatorial interactions of multiple factors which can prove to be beneficial to decipher complex clinical phenomenon such as AD which is otherwise difficult to explain using single biomarkers as highlighted by Henriksen et al. (2014).

To validate our model, we considered diagnostic classification of AD patients from normal control (NC) using ADNI dataset (http://adni.loni.usc.edu/). Experimental results show that our method can generate highly interpretable as well as accurate machine learning models which can serve as an indispensable tool in the era of precision medicine. By considering a hypothetical cost model, we have also shown how our method can lead to a cost-effective diagnosis framework. A Python implementation of SHIMR can be downloaded from GitHub (https://github.com/tsudalab/SHIMR).

## Methods

### Data

Data used in the preparation of this article were obtained from the ADNI database (adni.loni.usc.edu). The ADNI was launched in 2003 as a public-private partnership, led by Principal Investigator Michael W. Weiner, MD. The primary goal of ADNI has been to test whether serial MRI, PET, other biological markers, and clinical and neuropsychological assessment can be combined to measure the progression of mild cognitive impairment and early AD. The plasma protein data was obtained from “Biomarkers Consortium Plasma Proteomics Project RBM multiplex data,” which contains 190 proteins previously reported in the literature to be related to human pathogenesis. Other data (such as demographic, diagnosis, MMSE and UPENN CSF biomarkers) were collected from the ADNIMERGE R package (ADNIMERGE_0.0.1.tar.gz). All these data were downloaded from the ADNI web-site (http://adni.loni.usc.edu/) as of March 23, 2016.

### Model

First, we briefly review existing machine learning based approaches and highlight their limitation to generate an interpretable CAD model which motivated the design of SHIMR. Kernel based methods such as SVM have been widely used for last two decades in several machine learning applications (Schölkopf & Smola, 2002). In SVM, the decision function is defined as $f(x) = \sum\nolimits_{i = 1}^n {a_i}K(x,{x_i})$. Where, *K* is a called the kernel function which is in general a non-linear function (Gaussian, polynomial kernels etc.) used to measure the similarity between any training example *x* and a support vector *x_i_*. Neural networks (Haykin, 2001) have similar decision functions using different non-linear activation functions (logistic, tanh, etc.). Due to such a non-linear transformation, it is often possible to generate highly accurate learning model but at the expense of losing model interpretability (aka “black box” model). To this end, DTs are often used which are generally interpretable as well as highly accurate (Beerenwinkel et al., 2002). The “if-then-else” rules employ Boolean clauses with logical AND and NOT operators (∧, ¬) to constitute complex features. However, complex features in DTs have limited forms and boosting is often combined (Dietterich, 2000) to circumvent such limitations, but at the cost of losing interpretability. To address this limitation, SHIMR represents the decision function as a weighted sum of conjunction rules, each of which is the conjunction of one-dimensional intervals. A conjunction rule looks like *I*(1.0 *≤ x*_3_ *≤* 2.0) *I*(*x*_5_ *≥* 0.5) *I*(*x*_6_ *≤* 0.8), where *I*(·) refers to the indicator function.

The model generated by SHIMR consists of sets of independent “if-then” rules which are in general simple, concise and highly interpretable compared to decision list (Lakkaraju, Bach & Leskovec, 2016). This simple structure allows intuitive visualization shown in Fig. 2. Complexity of decision list (Blum, 1998; Klivans & Servedio, 2006; Valiant, 1999; Clark & Niblett, 1989) comes from the “if-then-else” clause of rule formation. Because of the “else” conjugate one needs to consider all the preceding rules that have already been turned out to be false to make a decision. SHIMR’s closest ancestor is itemset boosting (Saigo, Uno & Tsuda, 2007), but it has extended functionalities for medical diagnosis such as dealing with continuous attributes, rejection option, class imbalance (Veropoulos, Campbell & Cristianini, 1999), calibration and visualization.

Let ${\mathbb H} = \left\{ {{h_1}, \ldots ,{h_M}} \right\}$ denote the set of all possible conjunction rules, where each feature *x_i_* is divided into a fixed number of intervals. SHIMR learns the following decision function from data,

$$f(x) = \sum\limits_{j = 1}^M {a_j}{h_j}(x) + b.$$

where *x* is the feature vector of a patient, *a_j_* is a weight associated with *h_j_*, and *b* is the bias term. In learning from data $\{ {x_i},{y_i}\} _{i = 1}^n$, the following objective function is minimized with respect to *a_j_* and b.

$$\sum\limits_{j = 1}^M |{a_j}| + {C^ + }\sum\limits_{\{ i|{y_i} = 1\} } {\rm\phi} (f({x_i})) + {C^ - }\sum\limits_{\{ i|{y_i} = - 1\} } {\rm\phi} ( - f({x_i}))$$

where φ is a loss function explained in the next section, *C*^+^ and *C*^−^ are the regularization parameters for positive and negative classes, respectively. This is an extremely high dimensional problem, but at the optimal solution, there are only a limited number of non-zero weights due to L1-norm regularization. We employ the column generation method (Demiriz, Bennett & Shawe-Taylor, 2002) that starts from the optimization problem with no variables and gradually grows the problem by adding one variable in each iteration. For selecting a variable efficiently, weighted itemset mining (Uno, Kiyomi & Arimura, 2005) is used. Refer to the “Supplemental Methods” of Article S1 for details about the learning procedure.

Decision making from *f*(*x*) is affected by the cost of rejection. Rejection is not as bad as misclassification, but incurs some cost as we need a different means for final decision. Assuming that the cost of misclassification is one, let us define 0 *≤ d ≤* 0.5 as the cost of rejection. Bartlett & Wegkamp (2008) showed that, if *η*(*x*) = *P*(*y* = 1 | *x*) is the posterior probability of *x* being classified to the positive class, the following decision rule achieves the smallest expected cost,

$$f_d^*(x) = \left\{ {\matrix{\hfill { + 1} & {{\rm\eta} (x) > 1 - d} \cr \hfill 0 & \hfill {d \le {\rm\eta} (x) \le 1 - d} \cr \hfill { - 1} & {{\rm\eta} (x) < d} \cr}}\right.$$

We use the above rule after converting *f*(*x*) to the posterior probability via isotonic calibration.

### Loss function

**Figure 3:**
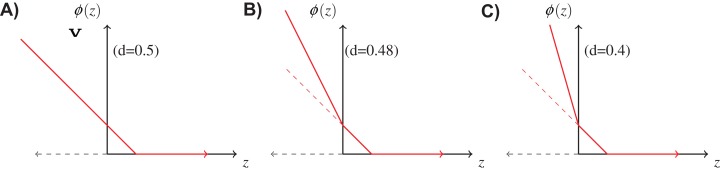
Double hinge loss function at different values of rejection cost *d*. (A) Double hinge loss at rejection cost, *d* = 0.5, (B) Double hinge loss at rejection cost, *d* = 0.48, (C) Double hinge loss at rejection cost, *d* = 0.4.

If the cost of rejection is known, it is reasonable to incorporate it in the loss function φ in the learning procedure. We use the following double hinge function proposed by Bartlett & Wegkamp (2008),

$${\rm\phi} (z) = \left\{ {\matrix{ 0 \hfill & {z \ge 1} \hfill \cr {1 - z} \hfill & {0 \le z < 1} \hfill \cr {1 - \displaystyle{{(1 - d)z} \over d}} \hfill & {z < 0} \hfill \cr} } \right.$$

where, $z = \sum\nolimits_{i = 1}^n {{y_i}f({x_i})} $ is the classification margin. Figure 3 shows the above function for different values of *d*. When *d* = 0.5, it is equivalent to the normal hinge loss. As *d* decreases, the slope in the negative domain becomes steeper, because the cost of misclassification becomes higher in comparison to *d*.

## Results

### Experimental settings

In this study we considered baseline concentration of plasma proteins of 151 subjects, out of which 97 subjects were diagnosed as AD patients and the remaining 54 subjects as NC. Here, we considered 14 proteins as the starting set of analytes. This is a collection of proteins responsible for the AD pathology as reported in literature (Signature #4; Llano, Devanarayan & Simon, 2013). The entire list of these 14 plasma proteins can be found in Table S1. We also considered baseline concentration of CSF and plasma proteins of 141 subjects from a common cohort to demonstrate a cost-effective diagnosis framework. Out of these 141 subjects, 88 subjects were diagnosed as AD and 53 subjects were diagnosed as NC. For CSF data we used tau, amyloid-β (Aβ) and phosphorylated tau (p-tau) proteins. We mainly used the ratio tau/Aβ and p-tau/Aβ as the features for CSF analysis. Baseline demographic information of all 151 subjects used in the current study is shown in Table 1. The entire dataset is divided into two stratified groups (two-third Train and one-third Test) using the same strategy as reported in Llano, Devanarayan & Simon (2013). Model generation and hyperparameters selection have been done using only the train dataset, whereas the unseen test data is used to report the classification test performance. To validate the performance of our method we have reported both internal cross validation as well as test results (Llano, Devanarayan & Simon, 2013). All the hyperparameters of the model are selected based on fivefold cross validation by running it 10 times. To report the internal cross validation performance, the training data has been divided into five stratified groups. At each iteration, four folds are used to generate the model, which is subsequently used to generate the results for the held out fold. This procedure is repeated for 10 times and the average results have been reported to minimize the data sampling bias. Classification performance has been evaluated using area under the receiver operating characteristics curve (AUC), accuracy (ACC), sensitivity (SN) and specificity (SP).

**Table 1:** Demographic information of 151 subjects from ADNI dataset used in this work.

Diagnosis	# of Subjects	Age	Gender (M/F)	Education	MMSE
AD	97	74.89 ± 7.97	53/44	15.21 ± 3.08	21.25 ± 4.62
NC	54	75.32 ± 5.84	28/26	15.6 ± 2.82	29.06 ± 1.21

**Notes:**

Standard deviations of variables Age, Education and MMSE scores are shown after “±” sign.

Abbreviations: AD, Alzheimer’s disease; NC, normal control; MMSE, Mini mental state examination.

### Interpretability vs. accuracy trade-off

**Figure 4:**
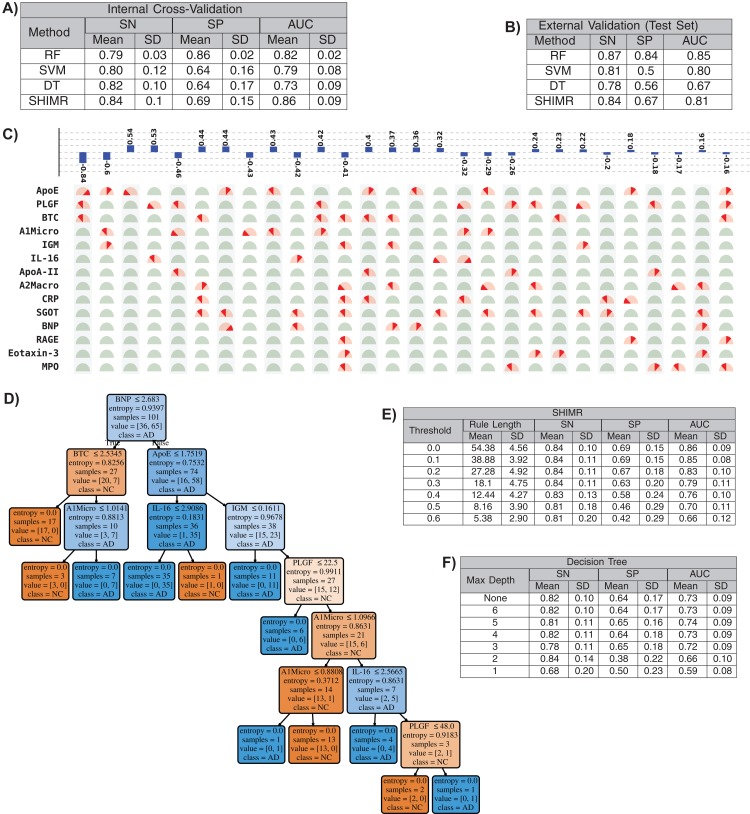
Interpretability vs accuracy trade-off. Comparison of classification (NC vs AD) performance between our method (SHIMR) at zero rejection rate (RR = 0) and other standard classifiers (RF, SVM and DT) both in terms of (A) internal cross validation and (B) external test set validation. Visual representation of the model generated by (C) SHIMR and (D) DT, respectively. (C) shows the weighted combination of simple rule based classification model generated by SHIMR and (D) shows long chains of conjugated rules generated by DT. Interpretability vs accuracy trade-off comparison between (E) SHIMR and (F) DT. A more interpretable model can be generated by tuning the weight threshold of features (in SHIMR) or controlling the maximum depth of tree (in DT) with a compromise in classification accuracy. Abbreviations: RF, random forest; SVM, support vector machines; DT, decision tree; SHIMR, sparse high order interaction model with rejection option; SN, sensitivity; SP, specificity; AUC, area under the curve; SD, standard deviation. Note: For results against RF, we have quoted the results taken from Llano, Devanarayan & Simon (2013). In SVM, we introduced non-linearity by using radial basis function kernel.

In this section we will present that our method has the ability to produce comparable accuracy as other existing non-linear methods without compromising the interpretability of the model. We will evaluate the interpretability of our SHIMR model both visually and quantitatively against another interpretable classification model, DT classifier. We will also present how the interpretability trades off classification accuracy and make a comparative study between SHIMR and DT. Figures 4A and 4B compare the performance of our method (SHIMR) against existing methods for AD vs NC classification. It can be observed that SHIMR can generate highly accurate classification models comparable to other existing non-linear models. The AUC of internal cross validation was 0.86 with a high sensitivity of 0.84 and reasonable specificity of 0.69. Next, we will show how our visualization module complements SHIMR by generating a simple and easily comprehensible visual representation of the model generated by SHIMR. Our visualization module can clearly represent the weighted combination of simple rules based classification model generated by SHIMR.

In Fig. 4C, the selected rules contributing to the model have been described as an intersection matrix, where each row represents individual feature (protein) and each column represents interaction among features (proteins) constituting a rule. A blue colored bar (above each column) shows the importance of each rule contributing to the overall model. Selected features are represented by “light red gauge” (semicircles), whereas the unselected one by “light green gauge.” The exact selected range of a particular feature is highlighted using “dark red wedge.” Looking in the clock-wise direction, each gauge represents the range of a particular feature where the left end represents the minimum and the right end represents the maximum. This representation highlights how interpretable our model is by clearly articulating the trained model in terms of the selected rules, rule importance and the attributes associated with each rule for individual subject. From Fig. 4C, one can understand that the generated rules are simple and easy to understand and possible to validate from domain knowledge. Whereas, DT classifier (Fig. 4D) generates a long chain of conjugated rules for the same classification task. Comparing Figs. 4A and 4B, one can see that SHIMR can also produce a better classification AUC (= 0.86) compared to DT (AUC = 0.73). To investigate how interpretability trades off the classification accuracy we further experimented with the feature importance threshold and maximum tree depth in SHIMR and DT, respectively, and the corresponding results are shown in Figs. 4E and 4F. From the results one can observe that a more interpretable model can be generated at the expense of classification accuracy. However, in case of SHIMR, it is possible to generate highly interpretable model (rule length 18) at a reasonable classification accuracy (AUC = 0.79) which is better than the accuracy (AUC = 0.73) of a full (not truncated) DT. To generate the full model as shown in Fig. 4C, SHIMR took 17.5–18 s on average, using standard MacBookPro laptop with Intel core i5, 2.9 GHz processor and 8 GB RAM.

### Interpretability vs. accuracy trade-off: comparing SHIMR with CORELS using plasma data

CORELS (Angelino et al., 2017) is a branch-and-bound optimization algorithm for finding optimal rule list from categorical data. CORELS leverages a number of theoretical bounds such as hierarchical objective lower bound, antecedent support lower bound, prefix length upper bound etc. to obtain optimal rule list more efficiently (both in terms of time and space requirements) than existing CART and other DT methods. We also compared the classification performance of SHIMR with that of CORELS using ADNI plasma data. Comparing the results of SHIMR (Table S5) with CORELS (Tables S3 and S4), it can be observed that CORELS can produce more interpretable model by generating less number of rules, but at the expense of losing classification accuracy. Accuracy of SHIMR (acc = 0.79) is much higher than the best achieved accuracy (acc = 0.69, λ = 0.02) obtained by CORELS. The details of parameter settings of CORELS and how it has been executed to generate the results of Tables S3 and S4 can be found in the “Supplemental Results” of Article S1.

## Discussion

### Adherence to the EU’s GDPR on algorithmic decision making and “Right to Explanation”

**Figure 5:**
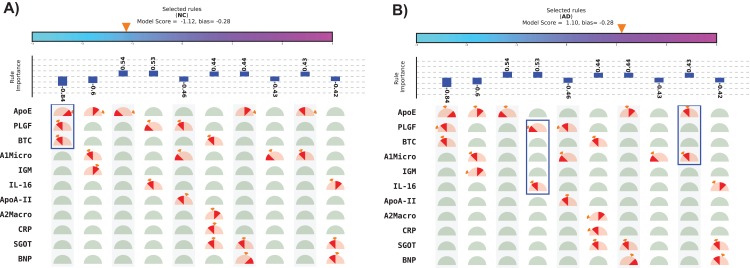
Visual representation of interpretable model generated for individual subject. (A) Normal control (NC) and (B) Alzheimer’s disease (AD). The top 10 rules have been depicted in this figure. The model score is generated as the sum of weights of the selected rules and bias. Abbreviations: NC, normal control; AD, Alzheimer’s disease. Note: The bias corresponds to the value of bias term of the model (for details refer to the “Methods” section). Here, we displayed only top 10 rules because of the space constraint. Therefore, sum of the weights of the displayed selected rules may not match the model score as in the case of (B). This will match exactly if the full model is displayed.

The visual representation of SHIMR can also help to explain a specific medical condition and its associated treatment in the context of precision medicine, as is evident from Figs. 5A and 5B. The blue rectangular box surrounding a set of proteins highlights the selected protein combination (or feature) for a subject. The “orange colored wedge” over each protein gauge describes the exact value of protein concentration corresponding to a particular subject. The generated model score for each subject is also highlighted by an orange pointer over a color bar at the top of each plot. From these figures one can understand that a high negative model score (−1.12) attributes to NC, whereas a high positive model score (1.10) attributes to AD and a model score close to zero corresponds to rejected (R) sample. Here, one can also identify the range of features attributed to AD, NC or R. Looking at such an interpretable CAD model, a doctor can understand the reason behind classification (NC or AD) as well rejection (R), and hence design a patient-specific diagnostic regime subsequently. Such a model is also helpful for a patient to understand his/her current medical condition, and hence, relate to the treatment prescribed by a doctor. Therefore, SHIMR can be considered to adhere to the EU’s GDPR on Algorithmic Decision Making and “Right to Explanation” (Goodman & Flaxman, 2016).

### Cost-effective framework

**Figure 6:**
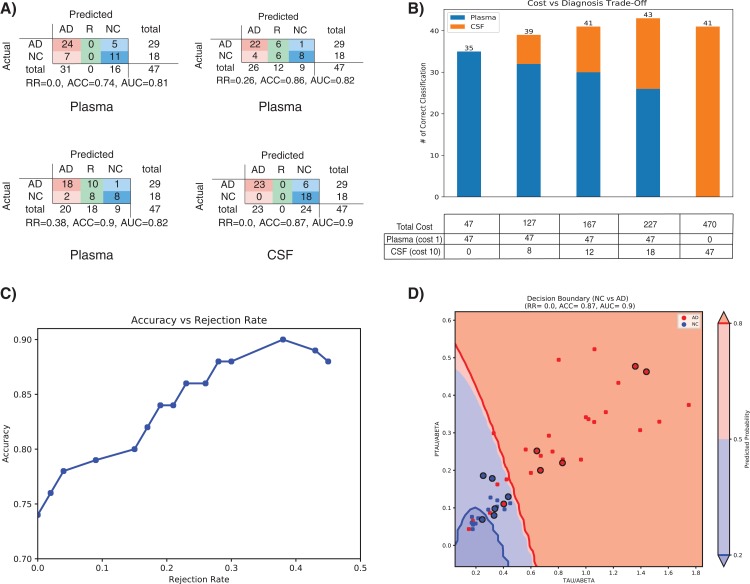
Effect of rejection option to design a cost-effective multistage diagnosis framework. (A) Model performance in terms of confusion matrix, accuracy (ACC) and area under the ROC curve (AUC) at different rejection rates (RR) for Plasma and CSF, respectively. (B) Trade-off between correctness of diagnosis and cost of screening. The numerical value below each bar plot refers to the total cost of screening and the numerical value above each bar plot represents the number of correctly diagnosed patients. The table below the bar plot describes the hypothetical cost of individual screening. The table cell entries along each row (Plasma or CSF) represent the cost of individual screening resulting in overall cost as represented by the numerical values below each bar plot. (C) Accuracy vs. Rejection Rate trade-off for SHIMR using plasma data. (D) A 2D decision boundary generated by SVM using CSF data. The samples clinically diagnosed as AD or NC are highlighted by red and blue dots, respectively. The samples rejected by SHIMR using plasma data are highlighted by drawing a circle around respective dots. Abbreviations: CSF, Cerebrospinal fluid; SVM, support vector machine; SHIMR, sparse high order interaction model with rejection option.

Treatment of AD is often hampered due to the lack of easily accessible and cost-effective biomarkers with reliable diagnostic accuracy as highlighted in the ‘Introduction’ section. In this section we will evaluate that how our method (SHIMR) can lead to an interpretable cost-effective multistage framework for clinical diagnosis by exploiting the notion of “classification with rejection option”. Here, we propose a cost-effective framework in the context of precision medicine. Figure 6A describes the effect of rejection for the classification of NC vs. AD using plasma and CSF data and how it can be exploited to design a cost-effective pathology for AD treatment. It can be seen that as the rejection rate is increased, the classification accuracy improves with increased prediction reliability (increased rejection rate infers higher decision threshold). Starting with an ACC of 0.74 at no rejection (RR = 0), it is possible to achieve a classification ACC = 0.9 at a higher rejection rate (RR = 0.38) using plasma data (Fig. 6A: Plasma). On the other hand, if CSF data is used for the same classification task, a more reliable prediction (ACC = 0.87) can be achieved with no rejection (Fig. 6A: CSF). Therefore, it can be argued that those 12 subjects (RR = 0.26) who are rejected using low-cost and easily accessible plasma biomarkers can now be recommended for a more sophisticated screening (e.g., CSF biomarkers). It can be observed from Table S2 that 11 out of those 12 subjects can be correctly classified using CSF data. Evidently, this highlights the efficacy of CSF screening in the current context. However, as highlighted before, it is not feasible to conduct CSF or other advanced screening for all registered patients due to the its invasive nature high financial burden or both. To visually depict this trade-off between correctness in diagnosis and cost of screening (Fig. 6B), we assumed a hypothetical cost model for plasma and CSF. Considering accessibility and invasiveness of the screening, we assumed the cost of one CSF screening as 10 unit if the cost of one plasma screening is one unit (10:1 ratio). With that assumption, it can be observed from Fig. 6B that instead of using CSF as the first stage screening which would otherwise cost 470 units for #of correct classification = 41, it would be more cost-effective to consider plasma at the first stage screening followed by CSF screening (cost = 167 units). Basically, the notion of applying rejection option can help a medical practitioner to decide on a systematic clinical regime for each patient individually, depending on the confidence of a machine learning model generated based on particular type of data. Hence, it is possible to start from a low-cost screening (e.g., plasma) followed by more sophisticated and invasive screening (e.g., CSF) for those patients only for which the model confidence is not so reliable. Hence, effectively controlling the rejection rate for a desired level of AUC (or accuracy) and subsequently applying good source of data, it is possible to design a patient-specific, cost-effective and reliable AD pathology, catering to the needs of both the patients and the health care system (Henriksen et al., 2014).

To understand how SHIMR internally works, the accuracy vs. rejection rate trade-off for the classification of NC vs. AD using plasma data has been depicted in Fig. 6C. It can be observed that as the rejection rate is increased, more and more data points which are close to the decision boundary and hence hard to classify get rejected and thus result in an improved accuracy after rejection. In order to understand how CSF data can be used to classify those rejected data points, a 2D decision boundary generated by SVM classifier for the same classification task has been plotted (Fig. 6D). The data points rejected by SHIMR using plasma have been highlighted by drawing a circle around respective data points on the same plot. In Fig. 6D, different decision confidence zones based on predicted probabilities have been highlighted using different colors. The dark red region represents high confidence zone for AD with positive predicted probability value more than 80% and dark blue region represents high confidence zone for NC with positive predicted probability value less than 20%. The region of intermediate positive predicted probability values are highlighted with light shades of respective colors. It is important to mention that SHIMR has the ability to identify the ambiguous low confidence zones (light red or blue) and refrain from taking any decision (reject) for those data points falling in that zone. Therefore, high rejection rate conforms to high prediction probability of the classified samples and hence more reliability in prediction. In a sense SHIMR makes decision only for those data points for which it is highly confident (high positive predictive probability) and thus can serve as a highly reliable CAD model to a medical practitioner (e.g., Doctor).

## Conclusion

To summarize, we have presented a highly accurate, interpretable and cost-effective machine learning framework in the context of precision medicine. We have formulated a sparse high order interaction model with an embedded rejection option and solved it using the simplex-based column generation method. The learning objective function is linear and convex, and hence it is possible to find a globally optimum solution. Our method can generate highly accurate and interpretable decision sets which are sets of “if-then” rules capturing the higher order interactions among a set of individual features. By embedding a rejection option and handling the class imbalance with separate misclassification costs for positive and negative examples, our method can judiciously mange the uncertainty of the traditional machine learning based model. To validate the effectiveness of our method, we conducted a diagnostic classification of AD from NC using an ADNI dataset and presented a cost-effective AD pathology. Our method performs feature selection and sample selection simultaneously and can lead to a very high accuracy: ACC = 0.9 (AUC = 0.82) at RR = 0.38 for AD vs. NC classification using plasma data. This potentially leads to a highly confident prediction model, a very desirable aspect in clinical diagnosis. We have shown that it is possible to design a patient-specific systematic multistage cost-effective AD pathology using a low-cost plasma profile followed by more advanced screening such as CSF. Large scale preventive care is possible by exploiting such patient specific machine learning framework which leverages low-cost and easily accessible plasma pathology as an early predictor of AD and subsequently recommends advanced pathology to those patients only for which it is not possible to generate desired level of accuracy using low-cost pathology. However, over-reliance on machine learning based automated diagnosis is a matter of concern as a single “False Negative” is highly expensive as it is associated with the life of the person being treated. The obvious social implication could be who or what would be made responsible for such misdiagnosis. Another concern is be related to the privacy and security of the patient data used for automated diagnosis. Automated diagnosis relies on electronic health records, the very construction of which may induce large and systematic mismeasurement, resulting in bias in automated diagnosis. Interpretable models such as SHIMR alleviate such concerns associated with automated diagnosis, but do not completely eliminate the role of a medical practitioner in medical diagnosis. A consensus among machine-derived diagnosis and diagnosis based on human expertise is desirable. Therefore, human intervention is inescapable in medical diagnosis where a doctor, the expert in this domain, can validate the automated diagnosis and use CAD as a helping hand and not as an entity of complete reliance.

## Supplemental Information

10.7717/peerj.6543/supp-1 Supplemental Information 1 The derivation of primal and dual objective functions along with the complete algorithm of SHIMR. Results of comparison of classification performance between SHIMR and CORELS using plasma data. Details of parameter settings of CORELS and how it has been executed to generate the results of Table S3 and Table S4.

10.7717/peerj.6543/supp-2 Supplemental Information 2 The list of starting set of 14 plasma proteins used in this work. This is a collection of plasma proteins responsible for AD pathology as reported in literature.

10.7717/peerj.6543/supp-3 Supplemental Information 3 Predicted diagnosis against the actual diagnosis using Plasma (RR=0.26) and CSF (RR=0.0) for individual patient (RID). RID represents the unique ID associated with each patient as defined in ADNI data set.

10.7717/peerj.6543/supp-4 Supplemental Information 4 Interpretability vs accuracy trade-off: CORELS with default parameters setting (-c 2 -p 1) on plasma data. The mean and standard deviation (SD) results of each performance metric (SN: Sensitivity, SP: Specificity and ACC: Accuracy) for five-fold cross validation are reported after running CORELS for ten iterations.

10.7717/peerj.6543/supp-5 Supplemental Information 5 Interpretability vs accuracy trade-off: CORELS with custom parameters setting (-c 3 -p 1 -a 1) on plasma data. The mean and standard deviation (SD) results of each performance metric (SN: Sensitivity, SP: Specificity and ACC: Accuracy) for five-fold cross validation are reported after running CORELS for ten iterations.

10.7717/peerj.6543/supp-6 Supplemental Information 6 Interpretability vs accuracy trade-off: SHIMR with different weight thresholds of features on plasma data. The mean and standard deviation (SD) results of each performance metric (SN: Sensitivity, SP: Specificity and ACC: Accuracy) for five-fold cross validation are reported after running SHIMR for ten iterations.

## Additional Information and Declarations

### Data Availability

The following information was supplied regarding data availability: GitHub: https://github.com/tsudalab/SHIMR.
