# Supplementary material for: An interpretable machine learning model for diagnosis of Alzheimer's disease
Source: PeerJ. 2019 Mar 1;7:e6543. doi: 10.7717/peerj.6543 (PMC6398390; doi:10.7717/peerj.6543)
Supplement: Supplemental Information 1 — Details of parameter settings of CORELS and how it has been executed to generate the results of Table S3 and Table S4. [file peerj-07-6543-s001.pdf]

Supplemental Article S1

Title: An interpretable machine learning model for diagnosis of Alzheimer's disease

Diptesh Das[1], Junichi Ito [2], Tadashi Kadowaki [2], Koji Tsuda[1,†]

[1]Department of Computational Biology and Medical Sciences, Graduate School of Frontier Sciences, The University of Tokyo, Chiba 277-8561, Japan
[2]Data Science Laboratory, hhc Data Creation Center, Eisai Co. Ltd., 5-1-3 Tokodai, Tsukuba 300–2635, Japan

[†]tsuda@k.u-tokyo.ac.jp

SUPPLEMENTAL RESULTS

To compare the classification performance of SHIMR with that of CORELS (Angelino et al. (2017)), we ran CORELS on the same data set of 14 plasma proteins used to generate the classification results by SHIMR (Table S5). We used 13,926 mined antecedents (binary features and conjunctions of binary features with minimum support 1 which is equivalent to normalized support of 0.01). The same minimum support = 1 (normalized support = 0.01) is used in case of SHIMR. First we ran CORELS with the default parameters which try to find optimal rule list by prioritizing several bounds and setting the symmetry-aware map flag to one (-p 1). The results are shown in Table S3. The mean and standard deviation (SD) results of each performance metric (SN: Sensitivity, SP: Specificity and ACC: Accuracy) for five-fold cross validation are reported after running CORELS for ten iterations. This default setting causes a heavy pruning of the search space to ensure high efficiency of the algorithm. Hence, with this default setting CORELS always ensures to generate minimum sized rule list even after tuning the regularization parameter to a small value (- r 0.005). Here one can observe that as we reduced the amount of regularization(r=0.03 $\rightarrow$ r=0.005), the accuracy dropped (acc=0.64 $\rightarrow$ acc=0.61) due to the effect of model overfitting.

In another setting, we ran CORELS to prioritize by the objective (-c 3) and at the same time we excluded the minimum support bounds (-a 1), but used the permutation map bound (-p 1). This allows CORELS to refrain from aggressive pruning based on several bounds. The results of this customized parameters setting are shown in Table S4. The mean and standard deviation (SD) results of each performance metric (SN: Sensitivity, SP: Specificity and ACC: Accuracy) for five-fold cross validation are reported after running CORELS for ten iterations. From Table S4, one can see that with this custom setting, CORELS captures more number of rules with increased classification accuracy. The best result is found for regularization parameter lambda=0.02. However, even with this custom setting, the CORELS could not capture more than 7 rules on average due to the upper bound of prefix length which is controlled by the current best objective and the value of regularization parameter lambda (for details please refer to the "Section 3.5 Upper bound on prefix length" of CORELS paper, Angelino et al. (2017)). As the lambda value is decreased, the classification accuracy drops owing to model overfitting. Now, comparing with the classification performance of SHIMR (Table S5), it can be observed that accuracy of SHIMR (acc=0.79) is much higher than the best achieved accuracy (acc=0.69, lambda=0.02) obtained by CORELS.

SUPPLEMENTAL METHODS

The loss function of SHIMR can be written as

$$\begin{aligned}\phi(z) &= max\{0,(1-z)\} + \frac{1-2d}{d}\max\{0,-z\} \\ &= (1-z)_+ + \frac{1-2d}{d}(-z)_+\end{aligned} \tag{1}$$

In terms of slack variables, the **learning objective** can be written as

$$\begin{aligned}&\underset{\xi_i,\gamma_i}{Minimize} \quad \phi(z) = \xi_i + \frac{1-2d}{d}\gamma_i, \\ &\qquad \text{Subject to} \quad \xi_i \geq (1-z_i) \quad \text{and} \quad \gamma_i \geq -z_i, \\ &\qquad \text{and} \quad 0.5 \geq d \geq 0\end{aligned} \tag{2}$$

Now handling the problem of class imbalance by choosing different regularization parameters separately for positive (C^+) and negative (C^-) classes, the primal objective of the above learning problem can be written as

Primal Objective:

$$\min_{a,b,\xi,\gamma} \quad \sum_{j=1}^{m}(a_j^+ + a_j^-) + C^+ \sum_{\{i|y_i=+1\}}^{n} \left(\xi_i + \frac{1-2d}{d}\gamma_i\right) + C^- \sum_{\{i|y_i=-1\}}^{n} \left(\xi_i + \frac{1-2d}{d}\gamma_i\right)$$

Such that

$$\begin{aligned}
& y_i\left(\sum_{j=1}^{m}(a_j^+ - a_j^-)H_{ij} + b\right) + \xi_i \geq 1, \quad \forall i = 1,\cdots,n \\
& y_i\left(\sum_{j=1}^{m}(a_j^+ - a_j^-)H_{ij} + b\right) + \gamma_i \geq 0, \quad \forall i = 1\cdots,n \\
& \xi_i \geq 0, \quad \gamma_i \geq 0, \qquad \forall \quad i = 1,\cdots,n \\
& a_j^+ \geq 0, \quad a_j^- \geq 0, \quad \forall j = 1\cdots,m
\end{aligned} \tag{3}$$

$H_{ij} = h_j(x_i)$ is the hypothesis of the ith sample, generated based on only the jth complex feature vector. Therefore, considering all the samples and all the feature vectors, the hypothesis matrix H can be represented as a n by m matrix, where each column $H_{\cdot j}$ is the output of the jth hypothesis on the training data. A typical hypothesis function can be defined as $H_{ij} = 2I(j \subseteq x_i) - 1$, where, $I(\cdot)$ refers to the indicator function. $I(\cdot) = 1$, if the condition inside holds true and 0 otherwise. Therefore, each hypothesis can assume either -1 or $+1$. Directly solving the primal objective is difficult due to large number of parameters. However, the dual objective can be solved efficiently using column generation based simplex method.

Dual Objective:

$$\begin{aligned}
W = \quad & \max_{P,Q} \quad \sum_{i=1}^{n} P_i \\
& \text{s.t.} \quad \left|\sum_{i=1}^{n}(P_i + Q_i)y_i H_{ij}\right| \leq 1, \quad \forall j = 1,\cdots,m \\
& 0 \leq P_i \leq C^+, \qquad \forall i = 1,\cdots,n|y_i = +1 \\
& 0 \leq P_i \leq C^-, \qquad \forall i = 1,\cdots,n|y_i = -1 \\
& 0 \leq Q_i \leq \frac{1-2d}{d}C^+, \qquad \forall i = 1,\cdots,n|y_i = +1 \\
& 0 \leq Q_i \leq \frac{1-2d}{d}C^-, \qquad \forall i = 1,\cdots,n|y_i = -1 \\
& \sum_{i=1}^{n}(P_i + Q_i)y_i = 0
\end{aligned} \tag{4}$$

The column generation based simplex method is an iterative procedure to find a subset $\hat{H}$ of the columns of H by using a base learning algorithm and then solve the restricted master problem until the optimum solution is reached. In each iteration the goal is to find the most violated constraint $|\sum_{i=1}^{n}(P_i + Q_i)y_i H_{ij}| \leq 1$ based on the current values of P and Q and add it to the hypothesis set to solve the restricted master problem. In our case, finding the most violated constraint corresponds to searching for a complex feature that maximizes the classification gain (g(j)=$|\sum_{i=1}^{n}(P_i + Q_i)y_i H_{ij}|$) as given in equation (5). To find the most violated complex feature (or hypothesis) in every iteration we used weighted LCM (Linear time Closed itemset Miner). Since our algorithm is a sparse convex linear optimization problem, global optimum solution is achieved. Our algorithm is summarized in Algorithm 1.

$$j^* \leftarrow \max_{j \in m}\left|\sum_{i=1}^{n}(P_i + Q_i)y_i H_{ij}\right| \tag{5}$$

Algorithm 1: SHIMR

1: Let, $u_i = P_i + Q_i$. Initialize $u \leftarrow (\frac{1}{n}, \cdots, \frac{1}{n})$
2: $d = 0.5$
3: **loop:**
4: $\{j\} \leftarrow$ LCM(u,s,X)
5: Find weak hypothesis (or feature set) using equation (5).
6: if($|\sum_{i=1}^{n}(P_i+Q_i)y_iH_{ij}| \leq 1$) then
7: break ▷ No more hypothesis
8: end if
9: $H \leftarrow H \cup \{j^*\}$ ▷ Update hypothesis
10: $(a,b,P,Q) \leftarrow$ solve (4) for a fixed value of 'd'.
11: s $\leftarrow max(g^+(j), g^-(j))$
12: **end loop**
13: Decrease $d(0.5 \geq d \geq 0)$ and repeat step (3).
14: Choose the best 'd^*' corresponding to the maximum gain,
$d^* \leftarrow \underset{d}{\text{argmax}}\ g(\{j\}) = \underset{d}{\text{argmax}}\ |\sum_{j=1}^{m}\sum_{i=1}^{n}(P_i+Q_i)y_iH_{ij}|$.
15: **return** (a^*, d^*).
16: end

Derivation of Dual:

The Lagrangian of the primal objective function can be written as

$$\begin{aligned}
\min_{a,b,\xi,\gamma} \quad L = \; & \sum_{j=1}^{m}(a_j^+ + a_j^-) + C^+ \sum_{\{i|y_i=+1\}}^{n}\left(\xi_i + \frac{1-2d}{d}\gamma_i\right) + C^- \sum_{\{i|y_i=-1\}}^{n}\left(\xi_i + \frac{1-2d}{d}\gamma_i\right) \\
& + \sum_{i=1}^{n}P_i\left[1 - \xi_i - y_i\left(\sum_{j=1}^{m}(a_j^+ - a_j^-)H_{ij} + b\right)\right] + \sum_{i=1}^{n}Q_i\left[-\gamma_i - y_i\left(\sum_{j=1}^{m}(a_j^+ - a_j^-)H_{ij} + b\right)\right] \\
& - \sum_{i=1}^{n}R_i\xi_i - \sum_{i=1}^{n}S_i\gamma_i - \sum_{j=1}^{m}T_j^+a_j^+ - \sum_{j=1}^{m}T_j^+a_j^- \\
= & \sum_{i=1}^{n}P_i + \sum_{j=1}^{m}a_j^+\left[1 - \sum_{i=1}^{n}(P_i+Q_i)y_iH_{ij} - T_j^+\right] + \sum_{j=1}^{m}a_j^-\left[1 + \sum_{i=1}^{n}(P_i+Q_i)y_iH_{ij} - T_j^-\right] \\
& - b\sum_{i=1}^{n}(P_i+Q_i)y_i + \sum_{\{i|y_i=+1\}}^{n}\xi_i\left[C^+ - P_i - R_i\right] + \sum_{\{i|y_i=-1\}}^{n}\xi_i\left[C^- - P_i - R_i\right] \\
& + \sum_{\{i|y_i=+1\}}^{n}\gamma_i\left[\frac{1-2d}{d}C^+ - Q_i - S_i\right] + \sum_{\{i|y_i=-1\}}^{n}\gamma_i\left[\frac{1-2d}{d}C^- - Q_i - S_i\right]
\end{aligned}$$

To solve the above minimization problem, we take the partial derivatives of L with respect to the variables a, b, ξ, γ.

$$\begin{aligned}
\frac{\partial L}{\partial a_j^+} = 0 \quad & \Rightarrow \quad 1 - \sum_{i=1}^{n}(P_i+Q_i)y_iH_{ij} - T_j^+ = 0 \\
\frac{\partial L}{\partial a_j^-} = 0 \quad & \Rightarrow \quad 1 + \sum_{i=1}^{n}(P_i+Q_i)y_iH_{ij} - T_j^- = 0 \\
& \Rightarrow \left|\sum_{i=1}^{n}(P_i+Q_i)y_iH_{ij}\right| \leq 1, \quad [T_j^+, T_j^- \geq 0], \quad \forall j = 1, \cdots, m
\end{aligned}$$

$$\frac{\partial L}{\partial b} = 0 \quad \Rightarrow \quad \sum_{i=1}^{n}(P_i+Q_i)y_i = 0$$

$$
\begin{aligned}
\frac{\partial L}{\partial \xi_i} = 0 \quad & \Rightarrow \quad C^+ - P_i - R_i = 0, \quad \forall i = 1, \cdots, n | y_i = +1 \\
& \text{and} \quad C^- - P_i - R_i = 0, \quad \forall i = 1, \cdots, n | y_i = -1 \\
& \Rightarrow \quad 0 \le P_i \le C^+, \quad [R_i \ge 0], \quad \forall i = 1, \cdots, n | y_i = +1 \\
& \text{and} \quad 0 \le P_i \le C^-, \quad [R_i \ge 0], \quad \forall i = 1, \cdots, n | y_i = -1
\end{aligned}
$$

$$
\begin{aligned}
\frac{\partial L}{\partial \gamma_i} = 0 \quad & \Rightarrow \quad \frac{1-2d}{d} C^+ - Q_i - S_i = 0, \quad \forall i = 1, \cdots, n | y_i = +1 \\
& \text{and} \quad \frac{1-2d}{d} C^- - Q_i - S_i = 0, \quad \forall i = 1, \cdots, n | y_i = -1 \\
& \Rightarrow \quad 0 \le Q_i \le \frac{1-2d}{d} C^+, \quad [S_i \ge 0], \quad \forall i = 1, \cdots, n | y_i = +1 \\
& \text{and} \quad 0 \le Q_i \le \frac{1-2d}{d} C^-, \quad [S_i \ge 0], \quad \forall i = 1, \cdots, n | y_i = -1
\end{aligned}
$$

Therefore, substituting the results of above partial derivatives, the dual objective becomes:

$$
\begin{aligned}
W = \quad & \underset{P,Q}{max} \quad \sum_{i=1}^{n} P_i \\
& \text{s.t.} \quad \left| \sum_{i=1}^{n} (P_i + Q_i) y_i H_{ij} \right| \le 1, \quad \forall j = 1, \cdots, m \\
& \quad 0 \le P_i \le C^+, \qquad \forall i = 1, \cdots, n | y_i = +1 \\
& \quad 0 \le P_i \le C^-, \qquad \forall i = 1, \cdots, n | y_i = -1 \\
& \quad 0 \le Q_i \le \frac{1-2d}{d} C^+, \qquad \forall i = 1, \cdots, n | y_i = +1 \\
& \quad 0 \le Q_i \le \frac{1-2d}{d} C^-, \qquad \forall i = 1, \cdots, n | y_i = -1 \\
& \quad \sum_{i=1}^{n} (P_i + Q_i) y_i = 0
\end{aligned}
$$

REFERENCES

Angelino, E., Larus-Stone, N., Alabi, D., Seltzer, M., and Rudin, C. (2017). Learning certifiably optimal rule lists. In *Proceedings of the 23rd ACM SIGKDD International Conference on Knowledge Discovery and Data Mining*, pages 35–44. ACM.
