# Supplementary material for: An interpretable machine learning model for diagnosis of Alzheimer's disease
Source: PeerJ. 2019 Mar 1;7:e6543. doi: 10.7717/peerj.6543 (PMC6398390; doi:10.7717/peerj.6543)
Supplement: Supplemental Information 2 — This is a collection of plasma proteins responsible for AD pathology as reported in literature. [file peerj-07-6543-s002.pdf]

Table S1: The list of starting set of 14 plasma proteins used in this work. This is a collection of plasma proteins responsible for AD pathology as reported in literature.

Abbreviation	Protein Name
**A1Micro**	**Alpha-1-Microglobulin**
**ApoAII**	**Apolipoprotein A-II**
**ApoE**	**Apolipoprotein E**
**BNP**	**Brain Natriuretic Peptide**
**SGOT**	**Serum Glutamic Oxaloacetic Transaminase**
**A2Macro**	**Alpha-2-Macroglobulin**
**BTC**	**Betacellulin**
**CRP**	**C-Reactive Protein**
**Eotaxin-3**	**Eotaxin-3**
**IGM**	**Immunoglobulin M (IGM)**
**IL16**	**Interleukin-16**
**MPO**	**Myeloperoxidase (MPO)**
**PLGF**	**Placenta Growth Factor (PLGF)**
**RAGE**	**Receptor for advanced glycosylation end**
