# Supplementary material for: An interpretable machine learning model for diagnosis of Alzheimer's disease
Source: PeerJ. 2019 Mar 1;7:e6543. doi: 10.7717/peerj.6543 (PMC6398390; doi:10.7717/peerj.6543)
Supplement: Supplemental Information 3 — RID represents the unique ID associated with each patient as defined in ADNI data set. [file peerj-07-6543-s003.pdf]

Table S2: Predicted diagnosis against the actual diagnosis using Plasma (RR=0.26) and CSF (RR=0.0) for individual patient (RID). RID represents the unique ID associated with each patient as defined in ADNI data set.

**RID**	**Diagnosis**		
	**Actual**	**Predicted**	
		**Plasma**	**CSF**
22	NC	Rejected	NC
91	AD	Rejected	AD
95	NC	Rejected	AD
120	NC	Rejected	NC
147	AD	Rejected	AD
177	NC	Rejected	NC
366	AD	Rejected	AD
426	AD	Rejected	NC
648	NC	Rejected	NC
931	NC	Rejected	NC
1161	AD	Rejected	AD
1262	AD	Rejected	AD
