# Supplementary material for: An interpretable machine learning model for diagnosis of Alzheimer's disease
Source: PeerJ. 2019 Mar 1;7:e6543. doi: 10.7717/peerj.6543 (PMC6398390; doi:10.7717/peerj.6543)
Supplement: Supplemental Information 4 — The mean and standard deviation (SD) results of each performance metric (SN: Sensitivity, SP: Specificity and ACC: Accuracy) for five-fold cross validation are reported after running CORELS for ten iterations. [file peerj-07-6543-s004.pdf]

Table S3: **Interpretability vs accuracy trade-off:** CORELS with default parameters setting (-c 2 -p 1) on plasma data.

**CORELS**								
**Lambda**	**Rule Length**		**SN**		**SP**		**ACC**	
	**Mean**	**SD**	**Mean**	**SD**	**Mean**	**SD**	**Mean**	**SD**
0.03	1.62	0.49	0.89	0.13	0.19	0.16	0.64	0.08
0.02	1.52	0.5	0.91	0.1	0.19	0.14	0.65	0.07
0.01	1.6	0.49	0.83	0.16	0.2	0.16	0.61	0.08
0.005	1.6	0.49	0.84	0.15	0.2	0.16	0.61	0.08
