# Supplementary material for: An interpretable machine learning model for diagnosis of Alzheimer's disease
Source: PeerJ. 2019 Mar 1;7:e6543. doi: 10.7717/peerj.6543 (PMC6398390; doi:10.7717/peerj.6543)
Supplement: Supplemental Information 5 — The mean and standard deviation (SD) results of each performance metric (SN: Sensitivity, SP: Specificity and ACC: Accuracy) for five-fold cross validation are reported after running CORELS for ten iterations. [file peerj-07-6543-s005.pdf]

Table S4: **Interpretability vs accuracy trade-off:** CORELS with custom parameters setting (-c 3 -p 1 -a 1) on plasma data.

**CORELS**								
**Lambda**	**Rule Length**		**SN**		**SP**		**ACC**	
	**Mean**	**SD**	**Mean**	**SD**	**Mean**	**SD**	**Mean**	**SD**
0.02	5.68	0.71	0.82	0.11	0.44	0.2	0.69	0.08
0.01	6.86	0.75	0.8	0.13	0.42	0.2	0.66	0.09
0.005	6.94	0.76	0.79	0.12	0.42	0.2	0.66	0.09
0.001	6.96	0.75	0.81	0.13	0.41	0.20	0.66	0.09
