# Supplementary material for: An interpretable machine learning model for diagnosis of Alzheimer's disease
Source: PeerJ. 2019 Mar 1;7:e6543. doi: 10.7717/peerj.6543 (PMC6398390; doi:10.7717/peerj.6543)
Supplement: Supplemental Information 6 — The mean and standard deviation (SD) results of each performance metric (SN: Sensitivity, SP: Specificity and ACC: Accuracy) for five-fold cross validation are reported after running SHIMR for ten iterations. [file peerj-07-6543-s006.pdf]

Table S5: **Interpretability vs accuracy trade-off:** SHIMR with different weight thresholds of features on plasma data.

SHIMR								
**Threshold**	**Rule Length**		**SN**		**SP**		**ACC**	
	**Mean**	**SD**	**Mean**	**SD**	**Mean**	**SD**	**Mean**	**SD**
0.0	54.38	4.56	0.84	0.10	0.69	0.15	0.79	0.09
0.1	38.88	3.92	0.84	0.11	0.69	0.15	0.79	0.07
0.2	27.28	4.92	0.84	0.11	0.67	0.18	0.78	0.08
0.3	18.1	4.75	0.84	0.11	0.63	0.20	0.76	0.08
0.4	12.44	4.27	0.83	0.13	0.58	0.24	0.74	0.08
0.5	8.16	3.90	0.81	0.18	0.46	0.29	0.69	0.10
0.6	5.38	2.90	0.81	0.20	0.42	0.29	0.67	0.10
